# Adjunctive Therapeutics in the Management of Cardiopulmonary Resuscitation: A Narrative Literature Review

**DOI:** 10.3390/jcm12237374

**Published:** 2023-11-28

**Authors:** Megan Hoffer, Robert C. F. Pena, Quincy K. Tran, Ali Pourmand

**Affiliations:** 1 Department of Emergency Medicine, School of Medicine and Health Sciences, George Washington University, Washington, DC 20037, USA; mhoffer@mfa.gwu.edu (M.H.); rcfp14@gwu.edu (R.C.F.P.); 2 Department of Emergency Medicine, School of Medicine, University of Maryland, Baltimore, MD 21201, USA; qktran@som.umaryland.edu; 3 Program in Trauma, The R Adams Cowley Shock Trauma Center, School of Medicine, University of Maryland, Baltimore, MD 21201, USA

**Keywords:** cardiac arrest, cardiopulmonary resuscitation, calcium, sodium bicarbonate, magnesium, steroids

## Abstract

Nearly 565,000 patients will suffer from prehospital and inpatient cardiac arrest in the United States per annum. Cardiopulmonary resuscitation and all associated interventions used to achieve it remain an essential focus of emergency medicine. Current ACLS guidelines give clear instructions regarding mainstay medications such as epinephrine and antiarrhythmics; however, the literature remains somewhat controversial regarding the application of adjunctive therapeutics such as calcium, magnesium, sodium bicarbonate, and corticosteroids. The available data acquired in this field over the past three decades offer mixed pictures for each of these medications on the effects of core metrics of cardiopulmonary resuscitation (e.g., rate of return of spontaneous circulation, survival-to-hospitalization and discharge, 24 h and 30 d mortality, neurological outcome), as well as case-specific applications for each of these interventions (e.g., polymorphic ventricular tachycardia, electrolyte derangements, acidosis, post-arrest shock). This narrative literature review provides a comprehensive summary of current guidelines and published data available for these four agents and their use in clinical practice.

## 1. Introduction

In the United States, 356,000 patients suffer from out-of-hospital cardiac arrest (OHCA) each year, presenting to the emergency department (ED) with an often-undifferentiated cause of arrest [1]. An additional 209,000 patients will then sustain in-hospital cardiac arrest (IHCA) after admission irrespective of their original diagnosis or involvement of cardiac arrest (CA) prior [2]. For the former, the challenge for emergency physicians is to attempt to reverse the underlying cause of arrest to restore circulation. For the latter, valuable data can still be derived for application in the ED.

A critical component of cardiac arrest management involves electrolyte shifts and related pathologic mechanisms. This can occur both as the possible cause of the initial arrest, as well as an insult acquired during the course of resuscitation, primarily due to acidosis, medication administration, and underlying comorbidities. Aside from standard medications such as epinephrine and antiarrhythmics, other adjunctive therapeutics, including calcium, magnesium, sodium bicarbonate, and corticosteroids, have been investigated to address this concern. Historically, these medications have been utilized due to theoretical benefits in reversing or mitigating physiological changes in cardiac arrest, yet the empiric and case-specific administration of each remains highly debated [1].

This review discusses the current clinical guidelines and available literature associated with each of these therapeutic interventions. Both general and specific outcomes have been examined by various researchers, including potential benefits in improving the achievement of return of spontaneous circulation (ROSC), effects on patient mortality, survival-to-hospitalization and discharge, neurological outcome, resolution of electrolyte derangements and concomitant acidosis, treatment of post-arrest shock, and related points of interest. Furthermore, the literature has attempted to demarcate particular circumstances for which certain adjuncts may provide optimal benefits, such as setting (out-of-hospital vs. inpatient), time to administration, and type of arrest (i.e., underlying rhythm or mechanism). This review aims to examine the available evidence regarding these adjunct medications to determine their effect on the achievement of ROSC during cardiopulmonary resuscitation.

## 2. Materials and Methods

The literature was evaluated through a preliminary search of two databases (PubMed, Scopus). Basic search terms including “cardiac arrest”, “resuscitation”, “calcium”, “magnesium”, “sodium bicarbonate”, “steroids”, “adjunct” and related terms were utilized. Two authors assessed each title and abstract independently according to the following criteria. Studies included randomized clinical trials, observational studies, and reviews. Expert opinion, commentary articles and editorials were excluded from this review. Search results were further limited to English language and those studies published before October 2023. If there was any discrepancy between two reviewers, they tried to resolve the difference by consensus before involving a third investigator. The process of reaching agreement after full-text review on eligibility for inclusion in the analysis was similar. The data were reported as a consensus between investigators, so no interrater agreement was calculated. We used the web-based Covidence system to manage our narrative review (www.covidence.org (accessed on 2 October 2023), Melbourne, Victoria, Australia).

## 3. Results

### 3.1. Calcium

Calcium has been frequently used during cardiac arrest due to the critical role the ion plays in myocardial contractility, and its hypothesized improvement on cardiac inotropy. Currently, the AHA guidelines on cardiac arrest recommend against the routine use of calcium, although the literature remains mixed [2] A randomized controlled clinical trial by Vallentin et al. (2021) [3] evaluated the administration of calcium vs. placebo in 397 patients during cardiac arrest. The trial was stopped early during interim analysis due to concern for harm in rates of achieving ROSC (19% vs. 27%; RR 0.72; 95% CI [0.49–1.03]; risk difference, −7.6%; 95% CI [−16%–+0.8%]; *p* = 0.09). Overall, 30 d survival was equivalent between groups (5.2% vs. 9.1%, RR 0.57; 95% CI [0.27–1.18]; risk difference, −3.9%; 95% CI [−9.4% to 1.3%]; *p* = 0.17) [3]. Vallentin et al. [4] also studied calcium in atraumatic, pulseless electrical activity (PEA) arrest in their Calcium for Out-of-Hospital Cardiac Arrest (COCA) Trial (n = 104), once again demonstrating reduced rate of ROSC (20% vs. 39%; RR 0.51; 95% CI [0.26–1.00]) and 30 d survival (2.2% vs. 13.6%; RR 0.16; 95% CI [0.02–1.26]) [4]. A meta-analysis from Kette et al. [5] revealed similar results, ultimately concluding calcium administration did not show any benefit in cardiac arrest, regardless of presenting rhythm [5].

Additionally, a recent observational analysis of pediatric cardiac arrest showed calcium conveyed no benefit in achieving sustained ROSC and was even associated with lower rates of survival-to-discharge (aOR 0.68; 95% CI [0.52–0.89]; *p* = 0.005) and lower rates of favorable neurologic outcomes (aOR 0.75; 95% CI [0.57–0.98]; *p* = 0.038) [6]. Another randomized controlled trial, again by Vallentin et al. [7], reports the same in adults, namely decreased rate of survival at one year and survival with favorable neurologic outcome (RR 0.42; 95% CI [0.18–0.97]) [7] However, one notable exception to these findings is the clear association between hypocalcemia and cardiac arrest itself, likely due to prolongation of the QTc interval [8,9]. One prospective observational study (n = 883) found high serum ionized calcium was associated with ROSC (OR 1.77; 95% CI [1.28–2.45; *p* = 0.001]), although it did not ultimately correlate with a survival benefit (OR 0.99; 95% CI [0.72–1.36]; *p* = 0.948) [10]. Cases of hyperkalemia, hypocalcemia, or hypomagnesemia, as seen in certain populations, may therefore benefit from calcium administration [11,12].

Conclusion: Evidence does not support the use of calcium in cardiac arrest, except in cases of severe hyperkalemia (see Table 1).

### 3.2. Magnesium

Magnesium administration in cardiac arrest has been evaluated in terms of both safety and efficacy for its use in cardiac arrest. The original 2015 American Heart Association (AHA) guidelines (and a recent 2018 update) recommend against its empiric use in cardiac arrest, although it does consider its potential efficacy specifically for QTc prolongation and resultant polymorphic ventricular tachycardia, otherwise known as torsades de pointes (TdP). Further research has also been conducted regarding its effects in refractory ventricular fibrillation (v-fib) arrest, broader applications of this electrolyte continue to be debated [18].

Magnesium was originally evaluated in a pilot study by Miller et al. [13] for safety as an ancillary therapy in cardiac arrest. They demonstrated no significant increase in patient harm and showed comparable rates of survival-to-discharge, as well as a temporal relationship between magnesium administration and ROSC [13]. Two early studies initially evaluated the potential efficacy of magnesium sulfate in both the prehospital and inpatient settings. The first was achieved through the Magnesium in Cardiac Arrest (MAGIC) Trial (1997) by Fatovich et al. [14] (n = 67), which determined early administration of MgSO_4_ (5 g IV) vs. placebo generated no significant difference in achievement of ROSC (23% vs. 22%) [19]. For inpatient arrest, Thel et al. [15] reported similar results, namely equivalent rates of ROSC (54% vs. 60%, *p* = 0.44), survival to 24 h (43% vs. 50%, *p* = 0.41), and survival-to-discharge (21% vs. 21%, *p* = 0.98) [15].

A randomized controlled, multicenter trial by Allegra et al. [16] evaluating epinephrine plus magnesium sulfate (2 g IV) vs. placebo, specifically in prehospital ventricular fibrillar arrest, again showed no significant difference in ROSC (25.5% vs. 18.%, *p* = 0.38), admission (16.4% vs. 16.7%, *p* = 1.0), or discharge (3.6% vs. 3.7%, *p* = 1.0) [16]. Shortly after, yet another trial conducted by Hasan et al. [14] (n = 105 patients) also did not find improved time to ROSC in ventricular fibrillation arrest after administration of magnesium (17% vs 13%; CI 95% [−10%–+18%]) [14] Moreover, evaluation in a randomized controlled trial by Longstreth et al. [17] and a retrospective analysis by Suzuki et al. [20] demonstrated no benefit in magnesium sulfate for neurologic outcome in patients with ventricular fibrillation arrest, although found no increased risk of harm [17,20]. Despite this, there have been several case reports describing ROSC after high dose magnesium administration in the specific instance of refractory vfib arrest [21].

Despite this, magnesium still serves an important role in pre-arrest and intra-arrest management as the treatment of choice for QTc prolongation and its subsequent degeneration into torsades des pointes [22]. The mechanism behind its utility in this instance is the theorized reduction in early after-depolarizations in cardiac myocytes to help terminate underlying dysrhythmias. Because of the same underlying mechanism of effect, it may be safely administered specifically in ventricular fibrillation arrest with profound electrolyte abnormalities (i.e., hypomagnesemia, hypokalemia) likely causing the arrest itself [23]. Although there are not many randomized controlled trials demonstrating this, it has been well-demonstrated in case reports and practice [24]. Per the updated 2018 European Resuscitation Council (and related) guidelines, magnesium sulfate is recommended in this specific instance, but not advised for empiric use in all cardiac arrests [25].

Conclusion: Magnesium should be given as a first-line agent in the management of torsades des pointes. Evidence has not shown benefit in other causes of cardiac arrest; however, there has been no harm demonstrated from administration (see Table 1).

### 3.3. Sodium Bicarbonate

Sodium bicarbonate (NaHCO_3_), considered to be a mainstay adjunct in resuscitative management, remains rather controversial. Typically administered as a buffer against systemic acidosis sustained during cardiac arrest, its empiric administration has remained a focus of discussion for decades. However, guidelines from the European Resuscitation Council from 2015 recommend against routine sodium bicarbonate use, except in cases of “life-threatening hyperkalemia, cardiac arrest associated with hyperkalemia,” and “cardiovascular toxicity (hypotension, cardiac arrhythmias) caused by tricyclic antidepressants and other fast sodium channel blockers” [26]. The American Heart Association (AHA) recommendations from the same year echo this, citing their original stipulation from 2010 that “routine use of sodium bicarbonate is not recommended for patients in cardiac arrest” despite the emergence of new research on the matter [27].

The literature indicates inherent potential in sodium bicarbonate in specific CA patient cohorts, although any reported benefits are largely drawn from retrospective data analyses and observational trials. Bar-Joseph et al. [28,29] performed a post hoc analysis of data derived from the Brain Resuscitation Clinical Trial III (BRCT III). In their first analysis (n = 2915, 54.5% NaHCO_3_), they noted a linear correlation between sodium bicarbonate and longer advanced cardiovascular life support (ACLS) duration [28]. Their second investigation examined 2122 OHCA patients with pre-ACLS downtime less than 30 min. In this case, the authors categorized treatment sites as “high users” or “low users” based on sodium bicarbonate administration. Here, “high user” locations sustained higher rates of ROSC (33.5% vs. 25.7%, aOR 1.36, 95% CI [1.08–1.70]), higher average rates of discharge (5.3% vs. 3.0%), and more favorable neurological outcomes (5.3% vs. 2.1%, aOR 2.18, 95% CI [1.23–3.86]). More interestingly, however, the authors note “high user” centers also more critically ill patients, including less “favorable” presenting rhythms (e.g., ventricular fibrillation, pulseless ventricular tachycardia), less patients with “short” time periods between collapse and arrest, and less bystander cardiopulmonary resuscitation (CPR). Despite this, such centers experienced better outcomes when these factors should have otherwise diminished or reversed such trends [29]. Contrary to this, a recent meta-analysis by Wu et al. [30] including six observational studies comprising 18,406 adult CA patients in Asia and North America determined no significant difference in ROSC (OR 1.185, 95% CI [0.680–2.065]) or survival-to-discharge (OR 0.296, 95% CI [0.066–1.323]) [30].

For out-of-hospital cardiac arrest (OHCA), to which the Emergency Medicine provider has greatest exposure and concern, Chen et al. [31] provides a retrospective, population-based cohort study of 5589 arrest patients. Their primary outcome of survival-to-admission was seen in 15.1% of all patients, while only 7.4% achieved their secondary outcome of 30 days, post-arrest survival. In the case of sodium bicarbonate, recipients experienced increased survival-to-admission in both general evaluation (aOR 4.47, 95% CI [3.82–5.22], *p* < 0.001) and propensity-matched analysis (aOR 4.61, 95% CI [3.90–5.46], *p* < 0.001), as well as improved 30 d mortality (88.1% vs. 94.8%, *p* < 0.0001) [31]. Similarly, a single-center, retrospective case-control study conducted in case-matched South Korean OHCA patients (n = 599, 2008–2013) showed increased ROSC in standard (OR 1.86, 95% CI [1.09–3.16], *p =* 0.022) and multivariate (aOR 2.49, 95% CI [1.33–4.65], *p* = 0.004) analysis. Additionally, patients were matched and stratified according to absolute dose administered (10–30 mEq/L cutoffs), which also showed significant improvement in the primary outcome (OR 1.18 per 20 mEq/L, 95% CI [1.04–1.33], *p* = 0.088; aOR 1.27, 95% CI [1.11–1.47], *p =* 0.001). The authors still support the routine use of sodium bicarbonate, citing its nature as a “last-ditch” as a factor driving its association with more negative outcomes [32]. To validate these results, Ahn et al. [33] conducted a small, double-blind, randomized, placebo-control trial involving 50 patients with sustained arrest (>10 min) and severe acidosis (pH < 7.1 or serum bicarbonate < 10 mEq/L). While confirming the significant effect of sodium bicarbonate on serum acidity (pH 6.99 vs. 6.90, *p =* 0.038) and bicarbonate deficit (21.0 vs. 8.0 mEq/L, *p =* 0.007), they found no improvement in rates of sustained ROSC (4.0% vs. 16.0%, *p =* 0.349) or good neurological outcome (0.0% vs. 4.0%, *p =* 1.000) [33].

In the inpatient setting, a single-center, retrospective, observational study by Wang et al. [34] assessed both sodium bicarbonate and calcium in cardiac arrest implicated by hyperkalemia and found similarly promising results. Their study cohort was small (n = 109 patients) and primarily focused on in-hospital cardiac arrests (IHCA) with confirmed hyperkalemia (K > 6.5 mEq/L). Here, they found 36.7% of patients regained spontaneous circulation and 3.7% survived to discharge. In the sodium bicarbonate treatment arm, ROSC was again significantly increased, particularly when serum potassium levels did not exceed 7.9 mEq/L (OR 10.51; 95% CI [1.50–112.89]; *p =* 0.03). The same could be said of those receiving calcium with potassium below 9.4 mEq/L (OR 51.11; 95% CI [3.12–1639.16]; *p =* 0.01) [34]. Contrary to this, a more recent retrospective cohort (n = 1060 patients) assessed by the same group only found improved neurological outcomes after treatment in sustained IHCA greater than 20 min. (OR 6.16, 95% CI [1.42–26.75]). Furthermore, greatest survival-to-discharge was seen in those without acidemia and not treated with sodium bicarbonate (OR 1.56; 95% CI [1.01–2.4]; *p =* 0.05). The authors therefore recommend against empiric administration and favor a case-specific approach (e.g., prolonged arrest) [35].

Another prospective, observational study by Kawano et al. [36] seems to challenge these results. Their study cohort of atraumatic arrest patients (n = 13,865), of which 37.3% were empirically administered sodium bicarbonate by paramedics in the field, experienced decreased rates of survival (2.3% vs. 19.8%) and favorable neurological outcome (1.2% vs. 10.6%) in the treatment arm. Further analysis confirmed the same after 1:1 propensity matching (aOR 0.64, 95% CI [0.45–0.91]; aOR 0.59, 95% CI [0.39–0.88]) and multiply imputed analysis (aOR 0.48, 95% CI [0.36–0.64]; aOR 0.54, 95% CI [0.38–0.76]). Despite these fewer promising results, the authors note substantially increased time from arrest to ROSC and higher-dose epinephrine requirements in the treatment cohort and the inherent selection bias this imparts [36].

The evidence therefore remains unclear as to whether sodium bicarbonate should be broadly employed for OHCA and IHCA. Yet as previously mentioned, the consideration of actual duration of arrest in the use of sodium bicarbonate remains open to debate. First proposed by Vukmir et al. [37] in a robust prospective, double-blinded, randomized clinical trial in 874 OHCA patients over 4 years, initial analysis did not demonstrate a significant difference in patient survival between the sodium bicarbonate (median 100.2 mEq/L, IQR 66.8–104.4) or placebo treatment subgroups (7.4% vs. 6.7%, *p* = 0.88). However, upon further evaluation, the authors demonstrated a near two-fold increase in survival in those who received treatment after prolonged cardiac arrest exceeding 15 min (32.8% vs. 15.4%, *p =* 0.007), thus raising the question of the utility of sodium bicarbonate in cardiac arrest of variable duration [37]. A subsequent retrospective study by Weng et al. [38] re-demonstrated this association of sodium bicarbonate and extended cardiac arrest (>15 min), although the achievement of ROSC was similar between the treatment and control arms after standard (40.0% vs. 32.3%, *p =* 0.465) and multivariate (aOR 1.270; 95% CI [0.501–3.219]; *p =* 0.615) analysis. Moreover, only 6.7% of patients who received sodium bicarbonate sustained ROSC and none survived to hospital discharge [38]. Vukmir et al. [37] note these poor outcomes may be partially attributed to the adverse side effects of sodium bicarbonate itself (e.g., hypernatremia, metabolic alkalosis, central venous acidosis with reduced exogenous pressor efficacy), although they warn of significant selection bias after eliminating all patients with shorter arrest times (<15 min), and therefore those with higher likelihood of positive outcomes [37,38].

The impact of sodium bicarbonate on patient outcomes such as the achievement of sustained ROSC and positive neurological outcome thus remain unclear. The current literature is highly retrospective in nature with few clinical trials available, although some do exist.

Conclusion: Evidence remains inconclusive, but does support the use of sodium bicarbonate in cases of severe acidosis or prolonged downtime without evidence of harm (see Table 2).

### 3.4. Corticosteroids

Steroids serve as yet another potential adjunctive therapeutic in cardiopulmonary resuscitation. Their utilization is based upon the premise that the hypothalamic-pituitary-adrenal (HPA) endocrine axis acts as a “self-defense” mechanism against stressors to homeostatic physiology, including myocardial infarction, sepsis, shock, surgical insult, and most severely, cardiac arrest itself. This results in endogenous release of HPA-controlled hormones including arginine vasopressin (ADH), adrenocorticotropic hormone (ACTH), and cortisol. One study by Ito et al. [39] comprising 36 cardiac arrest patients (nine survivors vs. 27 non-survivors) revealed globally elevated plasma levels of all three of these in both groups, presumably due to stress induced during arrest and subsequent resuscitation attempts. More interestingly, serum cortisol was significantly increased in survivors (*p =* 0.029), which allowed for derivation of an ROC curve (cutoff of cortisol 16.7 µg/mL) for predicting non-survival in this cohort (Sn 100%, Sp 100%, NPV 0.519) [39]. Since then, exogenous corticosteroids and vasopressin have been extensively utilized to replicate this physiological response in hopes of improving absolute mortality in cardiac arrest, as well as secondary post-arrest outcomes after initial ROSC is achieved.

Recent trials have yielded promising results regarding the use of single or multiple agents to achieve ROSC in both inpatient and out-of-hospital settings. One prospective, nonrandomized, pilot study by Tsai et al. [40] demonstrated significantly higher rates of ROSC in OHCA patients treated with hydrocortisone (100 mg IV) vs. placebo (61% vs. 39%, *p =* 0.038) with a markedly pronounced increase when administered within 6 min of ED arrival (90% vs. 50%, *p =* 0.045). However, 1 d and 7 d patient survival and hospital discharge rates were not different between subgroups, although the treatment arm did not experience worse rates of typically anticipated sequelae of stress-dose steroids (e.g., electrolyte disturbances, gastrointestinal bleeding, infection) [41]. Similarly, a recent Japanese retrospective study (n = 2223 patients) investigating hydrocortisone in both IHCA or OHCA not only reinforces this association of steroids and obtaining ROSC (25% vs. 8%, *p <* 0.001), but showed potentially increased survival-to-discharge as well (21% vs. 11%). The latter outcome deteriorates from significance (crude OR 2.2, 95% CI [1.12–3.97], *p =* 0.015; aOR 4.2, 95% CI [1.60–10.98]; *p =* 0.004) to non-significance after inverse probability treatment weighting (OR 3.34; 95% CI [0.88–13.44]; *p =* 0.077) and 1:1 propensity-score matching (OR 2.8; 95% CI [0.88–8.64]; *p =* 0.083). This was most likely due to the retrospective nature of the study and variable hydrocortisone dosing and administration of other adjuncts, as well as the number of patients used for the initial crude OR (n = 13 hydrocortisone vs. n = 240 non-hydrocortisone) [42]. In contrast, a subsequent, larger, retrospective, propensity score-matched Taiwanese study (n = 5445 patients) reported decreased mortality prior to discharge (75.65% vs. 80.86%; aHR 0.74, 95% CI [0.70–0.77]; *p <* 0.0001) and before 1 year (83.54% vs. 87.77%; aHR 0.73, 95% CI 0.70–0.76; *p <* 0.0001) in patients who received post-arrest steroid therapy [40].

Advancing beyond single therapeutic adjuncts, Mentzelopoulos et al. [43, 44] first demonstrated the benefit of the combined VSE regimen vasopressin (20IU IV), epinephrine (1 mg IV), and methylprednisolone (40 mg IV)) in a single-center, randomized, double-blind, placebo-controlled clinical trial. Patients received either VSE each resuscitation cycle or epinephrine with placebo (n = 48 treatment vs. 52 control), while those with post-resuscitation shock were also treated with stress-dose hydrocortisone (300 mg IV daily ×7 d maximum with gradual taper) vs. placebo (n = 27 treatment vs. 15 control). Those receiving VSE therapy experienced greater incidence of ROSC (81% vs. 52%, *p =* 0.003) and survival-to-discharge (19% vs. 4%, *p =* 0.02) with post-resuscitation shock patients receiving stress-dose steroids also experiencing improvement in the latter (30% vs. 0%, *p =* 0.02). Even more so, those completing full courses of hydrocortisone after initial shock benefited from more days free of all-organ, hepatorenal, respiratory, neurologic, and coagulation failure (*p =* 0.001–0.040) [43]. Several years later, the same Greek research group published their results from a similar randomized, placebo-controlled trial (n = 268 patients, three tertiary centers) examining the effects of the same therapies, only this time with further evaluation of neurological outcome. Here, patients in the treatment arm again experienced higher rates of ROSC (83.9% vs. 65.9%; OR 2.98, 95% CI [1.39–6.40]; *p =* 0.005) with survival-to-discharge with favorable neurological outcome in both these patients (13.9% vs. 5.1%; OR 3.28; 95% CI [1.17–9.20]; *p =* 0.02) and those with stress-dose steroid-treated post-resuscitation shock (21.1% vs. 8.2%; OR 3.74, 95% CI [1.20–11.62]; *p =* 0.02). In revisiting their pooled data from these two studies, the additional secondary outcome of lethal septic shock was assessed and found to be less likely in the VSE vs. placebo arm (HR 0.40–0.44; 95% CI [0.20–0.8]; *p =* 0.012–0.019) [44]. Finally, an additional randomized, double-blind trial conducted by Donnino et al [45]. also investigated post-resuscitation shock management with hydrocortisone (100 mg IV), but did not find significant differences in time to shock reversal (HR 0.83; 95% CI [0.40–1.7]; *p =* 0.63), shock reversal itself (52% vs. 60%, *p =* 0.57), good neurological outcome (24% vs. 32%, *p =* 0.53), or survival-to-discharge (28% vs. 36%, *p =* 0.54). However, further subdivision by baseline cortisol level < 15 µg/dL did reveal potential benefit from hydrocortisone in shock reversal (100% vs. 33%, *p =* 0.08), although not necessarily mortality (50% vs. 100%, *p =* 0.43) [45].

This work has since spurred further research into combination steroid-vasopressin therapy in resuscitation management and its subsequent effects on patient morbidity and mortality. Recently, Andersen et al. [46] conducted a large, multicenter, randomized, double-blind, placebo-controlled Danish clinical trial called the Vasopressin and Methylprednisolone for In-Hospital Cardiac Arrest (VAM-ICHA) Trial (n = 501 patients, 10 hospitals) to examine the effects of combined vasopressin (40IU IV) and methylprednisolone (40 mg IV) vs. placebo in IHCA. Patients treated with steroids and vasopressin experienced greater ROSC (RR 1.30; 95% CI [1.03–1.6]; *p =* 0.03), but again, saw no difference in overall 30 d survival (9.7% vs. 12.0%; RR 0.83; 95% CI [0.50–1.3]; *p =* 0.48) or favorable neurological outcome at 30 d and 90 d (7.6% vs. 7.6%; RR 1.00; 95% CI [0.55–1.83], *p* > 0.99) [46]. This was reconfirmed in subsequent analysis of long-term outcomes demonstrating no difference in survival (6.3% vs. 8.3%; RR 0.76, 95% CI [0.41–1.41]) or favorable neurological outcome (5.9% vs. 7.6%; RR 0.78, 95% CI [0.41–1.49]) after 6–12 months [47].

Conclusion: Steroids may provide an improvement in the achievement of ROSC, without clear benefit to overall mortality or survival to discharge. There has not been found to be evidence of harm, so corticosteroids may be considered as a safe adjunct therapy (see Table 3).

## 4. Discussion

In this review, we examine the literature surrounding four adjunct therapies and their roles in the management of cardiac arrest: calcium, sodium bicarbonate, magnesium, and steroids. These adjunct therapies are not included in the current ACLS algorithms but have been widely used by emergency physicians during cardiac arrest, and it is important to determine when they might be of benefit to selected patients and when they ought to be withheld.

Calcium chloride is typically supplied in code carts as an intravenous push dose for use in cardiac arrest, as an adjunct to epinephrine to improve myocardial contractility. However, the evidence presented in this review does not support its use except in cases of severe hyperkalemia. Several studies have actually shown risk of harm with empiric calcium administration in both pediatric and adult populations, including decreased rate of ROSC and hospital discharge. The one exception to this has been improvement in the rates of ROSC in cases of severe hyperkalemia, as calcium can stabilize the cardiac membrane in these patients (Table 1). Based on this evidence, it is not recommended to administer calcium routinely in cardiac arrest, unless hyperkalemia is confirmed as a cause using bedside electrolyte panels, or if the clinical history strongly supports this as a cause, such as in the case of patients who have missed hemodialysis sessions.

Magnesium is recommended as a first-line medication for polymorphic ventricular tachycardia, or torsades des pointes. It has also been studied as an adjunct therapy in cases of refractory ventricular fibrillation. Although shown to be of benefit in TdP, there has not been evidence to support its routine use for management of refractory ventricular fibrillation. However, unlike empiric calcium administration, there has been no evidence of harm in magnesium administration, and so this may be considered for use in cases with electrolyte abnormalities or undifferentiated cause of arrest without concern for harm to the patient (Table 1).

Sodium bicarbonate is commonly given as an adjunct therapy in cardiac arrest for its benefit in correcting severe acidosis, which decreases cardiac contractility, peripheral vascular tone, and beta receptor sensitivity to vasopressor medications. Although studied widely, the literature remains mixed. Sodium bicarbonate is notoriously difficult to study, in part due to its aforementioned status as a “last ditch effort” intervention for patients with more severe acidosis on presentation and prolonged resuscitation time, often the same patients who have worse outcomes due to underlying pathology at baseline. The evidence presented in this review (Table 2) remains inconclusive; however, the literature supports its use for patients with severe acidosis or prolonged cardiopulmonary resuscitation without evidence of harm.

Steroids have been given in cardiac arrest in order to support the physiologic stress response, with evidence showing higher levels of cortisol in survivors of cardiac arrest. Several large trials have suggested an improvement in ROSC in patients who receive steroids during cardiac arrest, with the largest improvements seen when steroids are given early in the resuscitation. The data to support whether steroids improve overall survival and mortality remains mixed, with some trials showing improvement in ROSC without improved survival to hospital discharge. None of the large trials have shown any evidence of harm with steroid administration, and it may be considered as a useful adjunct in resuscitation (Table 3).

## 5. Limitations

There are several limitations in our review. First, our review examines four adjunct therapies which are known to have mixed or inconclusive data despite being studied extensively. Although the purpose of this review is to provide better context for this mixed data, it will also make the review inherently prone to differences in conclusions and interpretation. Second, the nature of a narrative review carries an inherent risk of errors of objectivity and interpretation. We have, however, attempted to minimize this by providing as much objective data as possible to demonstrate how our conclusions and recommendations were drawn.

## 6. Conclusions

Based on our review, we recommend against the routine use of calcium in cardiac arrest, except in cases of severe hyperkalemia. Magnesium administration also does not seem to have clear benefit when administered, although it has not been found to cause harm and thus may be given empirically in certain patient subsets (i.e., electrolyte derangements, TdP). The literature regarding sodium bicarbonate remains unclear with current guidelines still recommending against its empiric use. It should still be considered, however, in those with severe acidosis and/or prolonged arrest. Intravenous corticosteroids and combination therapies (e.g., VSE) have been shown to have some benefit in cardiac arrest, with several large trials showing improvement in rates of ROSC, although this may not ultimately translate into improvement in overall survival. Their use in cardiac arrest and post-arrest shock may therefore be considered as safe and potentially advantageous.

## Figures and Tables

**Table 1:** Summary of available clinical data regarding the use of magnesium sulfate and calcium chloride in cardiopulmonary resuscitation.

Clinical Data for Adjunctive Electrolyte Therapeutics (Calcium and Magnesium) in Cardiopulmonary Resuscitation					
Electrolyte Treatment	Author (Year) [Location(s)]	Study Type (Size)	Description of Study	Results of Significance	Notes
**CALCIUM**	Vallentin et al. (2021) [Denmark] [3]	Randomized Controlled Trial (n = 391)	Calcium vs. saline placebo administration in OHCA	Calcium trending towards inferiority vs. placebo in ROSC (RR 0.72; 95% CI [0.49–1.03]; *p* < 0.9); equivalent 30 d mortality (RR 0.57; 95% CI [0.27–1.18], *p* = 0.17) and 30 d neurologic outcome (RR 0.48; 95% CI [0.20–1.12]; *p* = 0.12).	Trial did not reach predetermined sample size, stopped early due to concerns of harm in treatment arm.
	Vallentin et al. (2022) [Denmark] [4]	Randomized Controlled Trial (n = 104) “Calcium for Out-of-Hospital Cardiac Arrest (COCA)” Trial	Calcium vs. saline placebo administration in PEA OHCA	Calcium vs. placebo achievement of ROSC (RR 0.51; 95% CI [0.26–1.00]); 30 d survival (RR 0.16; 95% CI [0.01–1.26]).	Trial did not reach goal sample size. Subgroup analysis of effects of calcium on ROSC in patients with EKG changes concerning for hyperkalemia/ischemia. Extended analysis of original COCA Trial patients further reaffirmed trend towards poor outcomes in calcium-treated patients.
	Vallentin et al. (2022) [Denmark] [7]	Extended analysis of original Randomized Controlled Trial (n = 391)	Extended analysis of original COCA Trial patients at 6–12 mos	Calcium vs. placebo 12mo survival (RR 0.51; 95% CI [0.24–1.09]; 12mo favorable neurologic outcome (RR 0.42; 95% CI [0.18–0.97]).	
	Kette et al. (2013) [International] [5]	Systematic Review (10 articles)	Review of literature utilizing calcium alone or with other therapeutics to achieve ROSC in IHCA/OHCA	Multiple articles analyzed did not demonstrate clear benefits of calcium administration during cardiac arrest under various situations.	
	Cashen et al. (2023) [United States] [6]	Secondary analysis of observational data from the ICU-RESUScitation (ICU-RESUS) Project (n = 1100)	Calcium use during pediatric IHCA	Calcium administration had equivocal ROSC (aOR 0.87; 95% CI [0.61–1.24]; *p* = 0.445); worsened survival to discharge (aOR 0.68; 95% CI [0.52–0.89]; *p <* 0.005) and survival to discharge with favorable neurologic outcome (aOR 0.75; 95% CI [0.57–0.98]; *p* = 0.038).	ICU-RESUS Project was a “parallel, stepped-wedge hybrid cluster randomized trial comparing a CPR quality improvement bundle to usual care for its effect on survival outcomes” (Participation from 18 PICUs + pediatric CICUs in US from 2016–2021).
**MAGNESIUM**	Miller et al. (1995) [United States] [13]	Prospective Open-Label Pilot Study (n = 62)	Safety analysis of magnesium use in cardiac arrest	Magnesium vs. standard ACLS had equivalent rates of ROSC (35% vs. 21%, *p* = 0.21), survival to discharge (5.2% vs. 4.5%), and neurologic recovery (21% vs. 9%, *p* = 0.17).	Early pilot study with recommendations for trials to evaluate effects of magnesium on CA, earlier administration of drug. Authors strongly recommend continued adherence to ACLS protocols of that time.
	Fatovich et al. (1997) [Australia] [14]	Randomized Controlled Trial (n = 67)	Magnesium vs. placebo administration in OHCA	Magnesium had equivalent results in all major outcomes/criteria, including ROSC (23% vs. 22%), survival from ED (13% vs. 11%), survival to discharge (1% vs. 0%), number of cardioversion attempts (*p* = 0.37), additional resuscitation attempts (29% vs. 33%).	Authors reference Miller et al. [13] and comment on paucity of research regarding early administration of pharmacologics in ACLS protocol of the time.
	Thel et al. (1997) [United States] [15]	Randomized Controlled Trial (n = 156) “Magnesium in In-Hospital Cardiac Arrest (MAGIC)” Trial	Magnesium vs. placebo administration in ICHA	Magnesium vs. placebo without significant differences in ROSC (54% vs. 60%, *p* = 0.44), survival to 24 hrs (43% vs. 50%, *p* = 0.41), survival to discharge (21% vs. 21%, *p* = 0.98), GCS (median 15 for both groups).	Authors state: “Given the difficulties of carrying out clinical trials in patients in cardiac arrest, it is not surprising this trial is the first of any standard advanced cardiac life support medication in human beings.”
	Allegra et al. (2001) [United States] [16]	Randomized Controlled Trial (n = 116)	Magnesium vs. placebo administration in atraumatic, ventricular fibrillation arrest	Magnesium vs. placebo did not yield significant difference in ROSC (25.5% vs. 18.5%, *p* = 0.38), hospital admission (16.4% vs. 16.7%, *p* = 1.0), discharge (3.6% vs. 3.7%, *p* = 1.0).	Pooled data from four studies in addition to this one also demonstrated no improvement in survival after administration of magnesium.
	Hassan et al. (2002) [United Kingdom] [14]	Randomized Controlled Trial (n = 105)	Magnesium vs. placebo in refractory/recurrent ventricular fibrillation OHCA	Magnesium vs. placebo did not show difference in ROSC (17% vs. 13%; aOR 1.69, 95% CI [0.54–5.30]), discharge (4% vs. 2%).	Comments on difficulty of controlling prehospital administration of magnesium, benefits may be dose-dependent, and suggestion of a multicenter trial for further investigation of this drug.
	Longstreth et al. (2002) [United States] [17]	Randomized Controlled Trial (n = 300)	Administration of magnesium, valium, both, or placebo immediately post-ROSC to facilitate “patients awakening”	Treatments regimens (sole therapy, combined, placebo) did not demonstrate significant differences in percent of patients “awake” (30.7–46.7%) or “independent” (17.3–34.7%) within 3mos.	Factorial design to study effects of medication combinations on cardiac arrest sequelae. Magnesium and valium may need to be given as early as possible to be truly neuroprotective agents.

**Table 2:** Summary of available clinical data regarding the use of sodium bicarbonate as an adjunctive therapeutic in cardiopulmonary resuscitation of in-hospital and/or out-of-hospital cardiac arrest.

Clinical Data for Sodium Bicarbonate Administration in Cardiopulmonary Resuscitation					
Clinical Setting	Author (Year) [Location(s)]	Study Type (Size)	Treatment (% NaHCO_3_)	Results of Significance	Notes
**COMBINED**	Bar-Joseph et al. (2001) [United States] [28]	Post Hoc Analysis (BRCT III Trial) (n = 2915)	NaHCO_3_ vs. None NaHCO_3_ administration highly variable (see notes)	“No correlation…between…SB use and the pre-ACLS hypoxia times.” “Direct linear correlation…between…SB use and the duration of ACLS.”	Indirect post hoc analysis of BRCT III Trial (sodium bicarbonate administration was not primary outcome studied, administration optional). NaHCO_3_ admin. varied greatly b/w 16 sites (3.9%–98.3%). For 2005 analysis, sites either “high user” vs. “low user” based on NaHCO_3_ admin. (< or >50%) and time b/w epi and NaHCO_3_ admin. (< or >10 min.).
	Bar-Joseph et al. (2005) [United States] [29]	Post Hoc Analysis (BRCT III Trial) (n = 2122)		“High user” sites had increased ROSC (33.5% vs. 25.7%; aOR 1.36; 95% CI [1.08–1.70]), higher discharge (5.3% vs. 3.0%), more favorable neurological outcomes (5.3% vs. 2.1%; aOR 2.18; 95% CI [1.23–3.86]).	
	Wu et al. (2020) [International] [30]	Systematic Review, Meta-Analysis (n = 18,406)	Variable between studies analyzed	No significant difference in ROSC (OR 1.185, 95% CI [0.680–2.065]) or survival-to-discharge (OR 0.296, 95% CI [0.066–1.323]).	Authors note worse outcomes in N. American subgroup (despite similar arrest-to-BLS time) possibly d/t mixed OHCA/IHCA in N. American groups vs. almost-exclusive ED events in Asia.
**OUT-OF-HOSPITAL**	Chen et al. (2018) [Taiwan] [31]	Retrospective Study (n = 5589)	NaHCO_3_ vs. None (33.7%)	NaHCO_3_ associated with increased survival-to-admission in both general evaluation (aOR 4.47; 95% CI [3.82–5.22]; *p* < 0.001) and propensity-matched (aOR 4.61; 95% CI [3.90–5.46; *p* < 0.001]) analysis; improved 30 d mortality (88.1% vs. 94.8%, *p* < 0.0001).	
	Kim et al. (2016) [S. Korea] [32]	Retrospective, Case Control Study (n = 599)	NaHCO_3_ (cutoff pts 10–30 mEq/L) (100.0%)	NaHCO_3_ resulted in improved ROSC in standard (OR 1.86; 95% CI [1.09–3.16]; *p =* 0.022) and multivariate (aOR 2.49; 95% CI [1.33–4.65]; *p =* 0.004) analysis. Improved ROSC also seen after patients were matched and stratified by NaHCO_3_ dose received (OR 1.18 per 20 mEq/L, 95% CI [1.04–1.33], *p =* 0.088; aOR 1.27, 95% CI [1.11–1.47], *p =* 0.001).	
	Ahn et al. (2018) [S. Korea] [33]	Randomized Controlled Trial (n = 50)	NaHCO_3_ vs. Placebo (50.0%)	No improvement in sustained ROSC (4.0% vs. 16.0%, *p =* 0.349) or good neurological outcome (0.0% vs. 4.0%, *p =* 1.000).	This study examined pts w/prolonged CPR (>10 min.) and metabolic acidosis (pH < 7.1, serum bicarb. <10 mEq/L). This study also noted no reduction in hypercarbia from NaHCO_3_ after hyperventilation (20 bpm × 2 min.).
	Kawano et al. (2017) [United States, Canada] [36]	Prospective Observational Study (n = 13,865)	NaHCO_3_ vs. None (37.3%)	NaHCO_3_ associated with decreased rates of survival (2.3% vs. 19.8%) and favorable neurological outcome (1.2% vs. 10.6%). Same results after 1:1 propensity matching (aOR 0.64, 95% CI [0.45–0.91]; aOR 0.59, 95% CI [0.39–0.88]) and multiply-imputed analysis (aOR 0.48, 95% CI [0.36–0.64]; aOR 0.54, 95% CI [0.38–0.76]).	Authors acknowledge lack of neurological outcome data in nearly 44% of patients.
	Vukmir et al. (2006) [United States] [37]	Randomized Controlled Trial (n = 874)	NaHCO_3_ (1 mEq/kg) vs. Placebo (48.05%)	No difference in survival (7.4% vs. 6.7%; RR 1.0236; 95% CI [0.142–0.1387]; *p* = 0.88).	Trend towards improved outcome in prolonged arrest (>15 min) s/p NaHCO_3_ (2-fold survival increase) (32.8% vs. 15.4%, *p* = 0.007).
	Weng et al. (2013) [Taiwan] [38]	Retrospective Study (n = 92)	NaHCO_3_ (median 100.2 mEq) vs. No NaHCO_3_ (32.61%)	No difference in ROSC between NaHCO_3_ vs. non-NaHCO_3_ in standard (40.0% vs. 32.3%, *p* = 0.465) and multivariate (aOR 1.270; 95% CI [0.501–3.219], *p* = 0.615) analysis.	
**IN-HOSPITAL**	Wang et al. (2015) [Taiwan] [34]	Retrospective Observational Study (n = 109)	NaHCO_3_ +/− Ca vs. NaHCO_3_ vs. Ca vs. Neither (52.3% NaHCO_3_ + Ca, 29.4% NaHCO_3_)	Improved ROSC s/p NaHCO_3_ when serum K < 7.9 mEq/L (OR 10.51; 95% CI [1.50–112.89]; *p =* 0.03).	This study examined NaHCO_3_ and Ca for IHCA w/hyperkalemia (K > 6.5 mEq/L). Increased ROSC s/p Ca when serum K < 9.4 mEq/L (OR 51.11, 95% CI [3.12–1639.16], *p =* 0.01).
	Wang et al. (2021) [Taiwan] [35]	Retrospective Observational Study (n = 1060)	NaHCO_3_ vs. None (69.2%)	Improved neurological outcomes only found after treatment with sodium bicarbonate in those patients with sustained IHCA >20 min. (OR 6.16; 95% CI 1.42–26.75). Inverse relationship b/w acidemia (pH < 7.18) and the same outcome (OR 0.24; 95% CI [0.11–0.52]; *p <* 0.001).	Survival-to-discharge increased in patients who did not receive NaHCO_3_ nor experienced acidemia (pH > 7.18) (OR 1.58; 95% CI [1.01–2.41; *p* = 0.05).

**Table 3:** Summary of available clinical data for sole corticosteroid and combined regimen use in cardiopulmonary resuscitation and post-resuscitation shock.

Clinical Data for Corticosteroid Regimens in Cardiopulmonary Resuscitation					
Adjunctive Therapies	Author (Year) [Location(s)]	Study Type (Size/Setting)	Treatment	Results of Significance	Notes
**STEROID-ONLY REGIMEN**	Tsai et al. (2007) [Taiwan] [41]	Prospective, Nonrandomized, Open-Label Pilot Study (n = 97 OHCA)	Hydrocortisone 100 mg IV vs. Placebo	Hydrocortisone group had significantly higher rates of ROSC compared to control (61% vs. 39%, *p* = 0.038), especially if administered within 6 min. of ED arrival (90% vs. 50%, *p* = 0.045). No differences between groups regarding electrolyte disturbances, GI bleeds, infection (*p* = 0.335–0.89), 1 d or 7 d survival, hospital discharge rates.	Double-blinded, randomized controlled trials are difficult in time-sensitive, OHCA situations. Total cortisol levels were measured, although authors cite literature stating free cortisol levels are more indicative of adrenal function.
	Niimura et al. (2017) [Japan] [42]	Retrospective Study (n = 2233)		Hydrocortisone treatment vs. placebo had higher rates of survival to discharge (21.1% vs. 11.0%; aOR 4.2; 95% CI [1.60–10.98]; *p* = 0.004). Hydrocortisone was also an independent predictor of survival to discharge (HR 4.6, *p* < 0.001).	
	Tsai et al. (2019) [Taiwan] [40]	Retrospective Study (n = 4119)	High Steroid (>50 mg/d) vs. Low Steroid (≤50 mg/d) vs. No Steroid Groups	Nonsteroid groups with higher rates of in-hospital death (80.86% vs. 75.65%; aHR 0.74; 95% CI [0.70–0.77]; *p* < 0.0001). Steroid group had significantly lower 1 yr mortality rate (83.54% vs. 87.77%; aHR 0.73; 95% CI [0.70–0.76]; *p* < 0.0001).	“Steroid use during hospitalization was associated with survival to discharge, regardless of age, gender, underlying diseases…shockable rhythm, and steroid use prior to cardiac arrest.” Authors note most benefits seen in the low dose, rather than high dose steroid cohort, citing issues of rapid tapering and other factors.
	Donnino et al. (2016) [United States] [45]	Randomized Controlled Trial (n = 50)	Hydrocortisone 100 mg IV vs. Placebo (q8 hrs or until shock reversal)	Steroids vs. placebo experienced no difference in time to shock reversal (HR 0.83; 95% CI [0.40–1.75]; *p* = 0.63), shock reversal (52% vs. 60%, *p* = 0.57), good neurologic outcome (24% vs. 32%, *p* = 0.53), survival to discharge (28% vs. 36%, *p* = 0.54). Hydrocortisone vs. placebo groups with differences in shock reversal if cortisol level < 15 µg/dL (100% vs. 33%, *p* = 0.08), not death (50% vs. 100%, *p* = 0.43).	Significant improvement in shock reversal with hydrocortisone in patients with significant adrenal insufficiency was seen in this study and should be further investigated. Authors comment that increased post-CA IL-6 levels are associated with worse morbidity/mortality, here reduced after hydrocortisone, but without improvement in secondary outcomes (may be a correlative, rather than a causative).
**COMBINATION STEROID REGIMEN**	Mentzelopoulos et al. (2009) [Greece] [43]	Randomized Controlled Trial (n = 100)	Vasopressin 20IU + Epinephrine 1 mg per resuscitation cycle vs. Placebo (×5 cycles max) + Methylprednisolone 40 mg IV vs. Placebo (first cycle) + Hydrocortisone 300 mg IV vs. Placebo qd if post-ROSC shock present (7 d max w/taper)	Treatment arm experienced high rates of ROSC vs. placebo (81% vs. 52%, *p* = 0.003), improved survival to discharge (19% vs. 4%, *p* = 0.02). Treatment arm with post-ROSC shock experienced improved survival to discharge (30% vs. 0%, *p* = 0.02), hemodynamics, central venous oxygenation, and more organ failure-free days.	Authors note choice of methylprednisolone vs. hydrocortisone for initial vs. post-ROSC therapy given different effects on myocardial contractility, vascular/immune modulation. Less treatment vs. placebo patients needed additional epinephrine, potentially due to “more potentially reversible major disorders” and “more rapid and favorable response of more severely ill study group patients to a superior treatment.”
	Mentzelopoulos et al. (2013) [Greece] [44]	Randomized Controlled Trial (n = 268)		Treatment group vs. placebo experienced higher rates of ROSC ≥ 20 min. (83.9% vs. 65.9%; OR 2.98; 95% CI [1.39–6.40]; *p* = 0.005), survival to discharge (13.9% vs. 5.1%; OR 3.28; 95% CI [1.17–9.20; *p =* 0.02]. Treatment group with post-ROSC shock also experienced improved rates of survival to discharge (21.1% vs. 8.2%; OR 3.74; 95% CI [1.20–11.62]; *p* = 0.02), hemodynamics, central venous oxygenation, less organ dysfunction.	This trial mirrored a previous study by Mentzelopoulos et al. (2009) with a focus on neurologic outcomes. Methylprednisolone may augment vasopressor effects of vasopressin and epinephrine to improve pre-ROSC hemodynamics to better preserve/protect neurologic function (along with decreased duration of ALS itself).
	Andersen et al. (2021) [Denmark] [46]	Randomized Controlled Trial (n = 501 IHCA)	Methylprednisolone 40 mg IV + Vasopressin 20IU IV vs. Placebo (after epinephrine)	Treatment group vs. placebo had improved ROSC (42% vs. 33%; RR 1.30; 95% CI [1.03–1.63]; *p* = 0.03], equivalent 30 d mortality (9.7% vs. 12.0%; RR 0.83; 95% CI [0.50–1.37]; *p* = 0.48) and favorable neurologic outcome (7.6% vs. 7.6%; RR 1.00; 95% CI [0.55–1.83]; *p* > 0.99).	Trial was powered based on primary outcome of ROSC, so underpowered for secondary outcome of favorable neurologic outcome (smaller number of patients who achieve both). Many potentially-eligible patients were not included for various reasons, some able to be addressed.
	Granfeldt et al. (2022) [Denmark] [47]	Randomized Controlled Trial (n = 501 IHCA) “Vasopressin and Methylprednisolone for In-Hospital Cardiac Arrest (VAM-IHCA)” Trial		Intervention group vs. placebo groups experienced equivalent rates of survival at 1 yr (6.3% vs. 8.3%; RR 0.76%; 95% CI [0.41–1.41]) and neurologic outcome at 1 yr (5.9% vs. 7.6%; RR 0.78; 95% CI [0.41–1.49]).	Authors report need for discrimination between changes in treatment effect over time vs. overall survival given high rates of comorbidities and secondary causes of death in post-ROSC patients.

## Data Availability

The data presented in this study are openly available in PubMed and Scopus.
